# Evaluation of biocontrol efficacy of rhizosphere *Pseudomonas aeruginosa* for management of *Phytophthora capsici* of pepper

**DOI:** 10.1371/journal.pone.0309705

**Published:** 2024-09-20

**Authors:** Chenzhen Li, Xianghui Gao, Yunfeng Huo, Tahani A. Y. Asseri, Xueliang Tian, Kun Luo

**Affiliations:** 1 School of Aine Arts and Design, Huaihua University, Huaihua, Hunan, China; 2 Department of Plant Quarantine, Forest Pest and Disease Management & Quarantine Station of Linxia Gansu Province, Linxia, Ganshu, China; 3 College of Plant Protection, Hunan Agricultural University, Changsha, Huan, China; 4 Henan Institute of Science and Technology, School of Resource and Environment, Xinxiang, Henan, China; 5 Department of Biology, College of Science, King Khalid University, Abha, Saudi Arabia; Sakarya Uygulamali Bilimler Universitesi, TÜRKIYE

## Abstract

A significant population of biocontrol microorganisms resides in the rhizosphere of plants, which can be utilized for plant disease control. To explore the potential of rhizosphere soil microorganisms as biocontrol agents against pepper blight, a bacterial strain Pa608 was screened from rhizosphere soil of pepper and identified as *Pseudomonas aeruginosa* through morphological characteristics and 16S rRNA sequences. The result showed that the strain Pa608 demonstrated antagonistic activity against *Phytophthora capsici*, effectively suppressing mycelial growth. The potted experiment showed a high control efficacy of 88.0%. Remarkably, the strain Pa608 also reduced the disease index of pepper blight in the field, resulting in control efficiencies of 74.9%. Moreover, the strain Pa608 also enhanced pepper plant height and yield. GC-MS analysis revealed the production of numerous secondary metabolites by the strain Pa608, with α-pinene displaying potent anti-oomycete activity by inhibiting *P*. *capsici* growth. In conclusion, *P*. *aeruginosa* Pa608 exhibited high biocontrol activity against *P*. *capsici* and can be utilized for the management of *P*. *capsici* in pepper cultivation.

## Introduction

Pepper (*Capsicum annuum* L.), is widely cultivated for their culinary uses. However, their production is often hindered by biotic or abiotic factors. Among biotic factors, plant pathogens cause severe damage to peppers, especially *Phytophthora capsici*, which is a destructive soil-borne disease for pepper cultivation [1]. This pathogen infects pepper plants through asexually generated characteristic biflagellate, unicellular and motile zoospores from the lemon-shaped sporangia. The pathogenicity of *P*. *capsici* is distinct, and the pathogen shows resistance to multiple fungicide, making it challenging to control with traditional chemical methods [2]. In recent years, the environmental pollution and resistance issues associated with chemical pesticides have become more severe [3]. Biological control has emerged as a preferred strategy for sustainable agriculture due to its minimal environmental impact, long-lasting efficacy, and reduced risk of resistance development [4,5].

Several microbial species with biocontrol activity against *P*. *capsici* were utilized for controlling the pathogen. *Ochrobactrum pseudogrignonense* NC1 significantly inhibited the mycelial growth and zoospore production of *P*. *capsici* [6]. *Bacillus cereus* B1301 and *Chryseobacterium* sp. R98 had high biocontrol activity against *P*. *capsici* [7]. *Trichoderma aggressivum* f. europaeum, *T*. *longibrachiatum*, *Paecilomyces variotii*, and *T*. *saturnisporum* could be used to control of *P*. *capsici* in pepper [8]. The epiphytic yeasts from Piperaceae had multiple antagonistic mechanisms contained yeasts volatile organic compound production, hyperparasitism, and the production of β-1,3-glucanase enzyme [9]. Five *Trichoderma strains*, such as *T*. *harzianum*, *T*. *longibranchiatum*, *T*. *yunnanense*, *T*. *asperellum* (T2-10 and T2-31) and *Trichoderma* sp., were excellent potential agents for controlling *P*. *capsici* [10].

Pseudomonas species have been widely employed for the biological control of *P*. *capsici*. The biocontrol mechanisms of Pseudomonas encompass competitive exclusion for space and nutrients, siderophore production (iron chelation), secretion of catabolic enzymes and secondary metabolism products, and induction of systemic resistance in host plants [11–14]. For instance, *Pseudomonas otitidis* YJR27 and *P*. *putida* YJR92 can inhibit *P*. *capsici* and manage Phytophthora blight in pepper plants [15]. *P*. *plecoglossicida* YJR13 and *P*. *putida* YJR92 not only effectively hindered mycelial growth, zoospore germination, and germ tube elongation of *P*. *capsici* but also colonized pepper roots through cell motility, biofilm formation, and chemotaxis towards root exudates [16]. Additionally, Pseudomonas species strains markedly inhibited sporangia formation, zoospore release, and mycelial growth in liquid culture [17].

Beneficial biocontrol bacteria are abundant in the rhizosphere of plants, aiding in plant resistance against pathogenic infections, enhancing nutrient absorption, and promoting plant growth [18]. To screen for biocontrol bacteria with plant growth-promoting attributes and to obtain low molecular weight substances from bacteria for controlling *P*. *capsici*, we tested a strain of *P*. *aeruginosa* isolated from the rhizosphere of pepper. In this study, the strain of *P*. *aeruginosa* demonstrated significant biocontrol efficacy against *P*. *capsici* both *in vivo* and *in vitro*. Moreover, the *P*. *aeruginosa* easily colonized to rhizosphere of pepper and highly suppress pepper blight in filed. Notably, the α-pinene produced by *P*. *aeruginosa* exhibited anti-oomycete activity. These findings offer a promising avenue for developing novel methods to prevent pepper blight caused by *P*. *capsici*.

## Materials and methods

### *P*. *capsici* isolation and identification

*P*. *capsici* was isolated from a diseased pepper plant with blight, cultured on potato dextrose agar (PDA) medium at 28°C, and identified through PCR amplification utilizing primers (ITS1 and ITS4) targeting the internal transcribed spacer sequence [19].

### Isolation and screening for biocontrol bacteria

The procedure followed the method with slight modifications [20]. For the isolation of biocontrol bacteria, the surface soil was first removed, followed by collection of soil from a depth of 5–10 cm in the rhizosphere of 10 pepper plants heavily affected by *P*. *capsici* in Gaoqiao Town, Changsha County, Hunan Province (113.33°E, 28.44°N). Ten-gram soil samples were suspended in 90 ml of sterile water, agitated for 30 minutes, and then serially diluted 10,000 times. Subsequently, 100 μl of the suspensions with the highest dilution were spread on Luria-Bertani agar (LB) plates and incubated at 28°C for 48 hours until bacterial colonies appeared. Single colonies were then selected, purified on LB agar plates for 3 days, and subsequently cultured in 500 ml liquid LB medium for 48 hours.

To screen for biocontrol bacteria, a 5 mm diameter *P*. *capsici* disc was placed at the center of a PDA plate. Next, 5 μl of each of the four bacterial suspensions were inoculated 2 cm away from the disc in a crisscross pattern on the agar plate. The plates were then incubated for 7 days to observe the inhibition of mycelial growth. Bacteria exhibiting anti-oomycetes activity were chosen for further validation.

To further validate the biocontrol activity, the candidate bacterium was streaked horizontally on the left of a PDA medium and incubated at 28°C for 48 h to obtain bacterial growth. After bacterial growth, a 5 mm diameter disc of *P*. *capsici* was placed on the right of the same plate. The bacterium was 2cm from the disc of *P*. *capsici*. Plates with both bacteria and *P*. *capsici* were incubated for at 28°C. Each treatment repeated 3 times. When mycelium*s of P*. *capsici* on the right of the plate (no bacterium) grow up to the edge of plate, the biocontrol activity of the bacterium was assessed comparing the inhibition of mycelium expansion in the presence of the bacterium strain, and measuring the mycelium radius in the direction of the bacterium. For each plate we calculated the average radius of the mycelia using the following formula: the rate of inhibition of mycelium growth = (Rb-Rc) / Rb, where Rb was the mycelium*s* radius in the opposite direction of the bacterium; where Rc was the mycelium radius in the direction of the bacterium. The bacterium with the highest anti-oomycete activity was analyzed further.

### Taxonomic identification of strain Pa608

#### Morphological characters and 16S rRNA sequencing

A bacterial strain exhibiting the highest anti-oomycete activity was designated as Pa608. The purified bacterium was cultivated on nutrient agar (NA) and LB medium for 3 days at 28°C, followed by an assessment of colony morphology and color. The Gram characteristics of the strain Pa608 were determined using a Gram stain kit. Subsequently, the shape and size of the strain Pa608 were examined through scanning electron microscopy.

For the molecular identification of the strain Pa608, colony PCR was employed to amplify the 16S rRNA sequences using primers 27F and 1492R [21]. The 20 μL reaction mixture contained approximately 50 ng of total DNA, 5 mM each of dNTPs, 20 pmol each of both forward and reverse primers, and 0.5 U of Taq DNA polymerase (TransGen Biotech Co., Ltd., Beijing, China). PCR amplification was performed in a thermocycler applying the conditions: Denaturation for 1 min at 94°C; Annealing for 45 sec at 56°C; Extension for 1 min at 72°C; Final extension for 10 min at 72°C. The PCR products were visualized through agarose gel electrophoresis and then sequenced by Beijing Qingke Biotechnology Co., Ltd. The obtained sequences were manually curated and compared against the National Center for Biotechnology Information (NCBI) database using BLASTn to identify the most closely related bacterial species. A phylogenetic tree encompassing the strain Pa608 and seven other bacteria within the genus *Pseudomonas* was constructed using the neighbor-joining algorithm with 1000 bootstrap replicates in MEGA7 [22].

#### Extracellular enzyme characteristic

Protease, cellulase, amylase and phosphate solubility activities of the strain Pa608 were assessed on LB agar medium supplemented with 3% skim milk powder, carboxymethyl cellulose agar medium (K₂HPO₄ 2.5g, Na₂HPO₄ 2.5g, Carboxymethylcellulose sodium 20.0g, Peptone 2.0g, Yeast Extract 0.5g, Agar 14.0g), 1% starch-pancreatic soy agar (Trypticase 15.0g, Enzymatic digest of soybean meal 5.0g, NaCl 5.0g, Soluble Starch 3.0g; Agar 15.0g), and a phosphate solubilization medium (Glucose, 10.0 g; KH_2_PO_4_, 10.9 g; (NH_4_)_2_SO_4_, 1.0 g; MgSO_4_•7H_2_O, 0.16 g; FeSO_4_•7H_2_O, 0.005 g; CaCl_2_•2H_2_O, 0.011 g; MnCl_2_•4H_2_O, 0.002 g; Agar 14.0g), respectively. The plates were incubated for 3 days at 28°C, and the characteristics of extracellular enzymes were investigated by measuring the transparent zones.

#### Inhibition effect of strain Pa608 on pathogens

The antimicrobial spectrum of the strain Pa608 was assessed against some plant pathogens, including *Sclerotinia sclerotiorum*, *Pyricularia oryzae*, *Diaporthe citri*, *Botrytis cinerea*, *Fusarium graminearum*, and *Penicillium simplicissimum*. The confrontational culture method, as described earlier, was used to determine the width of the inhibitory zone, which was observed and quantified.

#### Pot experiment

The pepper variety Zhongke M105 f1 was selected for the pot experiment. The pepper seeds were disinfected with 0.1% sodium hypochlorite and thoroughly rinsed with distilled water three times to remove any sodium hypochlorite residue. The treated seeds were then placed on filter paper in a petri dish until germination occurred. Once sprouts emerged, they were transplanted 2 cm deep into soil in a 10-cm wide pot, with one plant per pot. The planting medium consisted of a 2:1 mixture of Walga horticultural nutrient soil and vermiculite. Plants were allowed to grow to the eight-leaf stage, and only robust and uniformly developed plants were selected for subsequent experiments.

To prepare the sporangia solution, *P*. *capsici* was first inoculated on oatmeal agar and incubated at 25°C for seven days, then transferred to 28°C for 48 hours under continuous illumination to induce sporangia formation. Subsequently, the sporangia were harvested from the agar surface using a brush [23]. The collected culture was then left at room temperature for three hours to encourage zoospore release, and subsequently diluted with distilled water to achieve an inoculation concentration of 2.0×10^4^ zoospores/ml. For the strain Pa608, inoculation was carried out in 100 mL LB medium and incubated at 28°C, 150 rpm, for 24 hours to achieve bacterial suspensions with a concentration of 1.0 ×10^8^ cells/ml, measured by a spectrophotometer.

Three treatments were implemented in the experiment. Treatment 1: Inject 3 ml of sterile water as a control. Treatment 2: inject 3 mL of Phytophthora zoospore suspension (2.0×10^4^ zoospores/ml) 1.5 cm deep into the soil [15], maintaining a distance from the plants to avoid direct contact. Treatment 3: Inject 3 mL of the strain Pa608 bacterial suspension (concentration: 1.0×10 ^8^ cells/ml) into the soil followed by 3 mL of Phytophthora zoospore suspension (2.0×10^4^ zoospores/ml). Each treatment contains 12 pots of peppers, with 3 pepper plants in each pot. Water regularly, maintain high temperature and humidity to promote disease development.

Disease severity (DI) was evaluated ten days post-inoculation utilizing a rating scale ranging from 0 to 5 [24], and the DI was determined using the formula [25]:

DI=(Σ(s×n)/(N×S))×100

where DI represented the disease index, s denoted the scale rating, n was the number of plants at a specific scale rating, N was the total number of evaluated plants, and S was the maximum scale rating.

#### Colonization dynamics of the strain Pa608 in pepper rhizosphere soil

Total of 6 pots of pepper plants were divided into 2 treatments, with 3 plants in each treatment. Treatment 1 involved injecting 3 mL of Phytophthora zoospore suspension (2.0×10^4^ zoospores/ml) into the soil at the base of the pepper plants [15], followed by 3 mL of the strain Pa608 bacterial suspension (1.0 ×10^8^ cells/ml). Treatment 2 involved injecting 3 mL of the strain Pa608 bacterial suspension into the soil at the base of the pepper plants. Soil samples from the pots were collected on days 1, 3, 5, 10, 15, 30, and 45. The soil samples were subjected to gradient dilution method to detect the quantity of the strain Pa608. Pseudomonas agar medium was used as the culture medium [26], diluted by a factor of 1, 000, and after incubating at 30°C for 2 days, the colonies were observed for color (blue-green) and counted.

#### Field experiment

The pepper greenhouse experiment was being conducted at the Vegetable Research Institute base of Hunan Agricultural Sciences Academy in Gaoqiao Town, Changsha County, Hunan Province. In previous years, the greenhouse has suffered from severe disease outbreaks, with an incidence rate of over 90%. The ridges were 1.2 meters wide, with 2 rows per ridge, a plant spacing of 35 cm, and a furrow width of 30 cm. There were 2 treatments: Treatment 1 was the blank control, with each pepper plant root irrigated with 50 mL of sterile water; Treatment 2 involved irrigating each pepper plant root with 50 mL of the strain Pa608 bacterial suspension (1.0 ×10^8^ cells/ml). The pepper seedlings were treated 3 days after transplanting, followed by two additional treatments in early June and early July, totaling 3 treatments. Harvesting and recording of plant height, yield and disease incidence were scheduled for July 28th. The disease index was classified according to Sunwoo’s standards as previously mentioned [25].

#### GC-MS analysis of the metabolites produced by the strain Pa608

The fermentation broth of the strain Pa608 was extracted using ethyl acetate (48 hours of fermentation). The ratio of organic solvent to fermentation broth was 1:1, with an extraction time of 8 hours, repeated three times. The extract was concentrated to a paste at 40°C using a rotary evaporator and stored at 4°C for future use.

The gas chromatography-mass spectrometry (GC-MS) was used to analyze the compound composition of the fermentation broth. The GC-MS analysis conditions were selected following the method [27], with adjustments made to the column temperature ramping up to 300°C at a rate of 10°C/min, hold for 40 min, using helium as the carrier gas with a split ratio set at 20:1, inject 1 μL, and set the injector temperature at 325°C. The mass spectrometry analysis conditions followed the method [27], with adjustments to stabilize the ion source at 280°C and scan the range from 33 to 600 m/z. The components of volatile compounds were identified by comparing retention times using the NIST mass spectral library. The relative contents were determined based on the percentage of peak areas of different compounds. A structural analysis of volatile organic compounds with antimicrobial potential was conducted based on relevant literature. The data were analyzed using the NIST Mass Spectral Library version 8 (NIST08.L) to match the acquired mass spectra, qualitatively analyze compounds based on matching factors, and retrieve pertinent information for each compound.

#### Biocontrol activity assessment of metabolites produced by the strain Pa608

The compounds with significant peak areas from the GC-MS results, particularly those previously recognized for their antimicrobial properties, were chosen as potential anti-oomycete substances. The pure form of these selected compounds was acquired and their inhibitory activity against *P*. *capsici* was evaluated using the growth rate inhibition method. The final concentrations of the candidate compounds in the PDA medium were 250 mg/L, 50 mg/L, 10 mg/L, and 5 mg/L, with an equal volume of sterile water serving as a control. One 5 mm diameter disc of *P*. *capsici* was positioned at the center of each petri dish and then incubated at a constant temperature of 25°C for 7 days. The procedure replicated three times. The inhibitory rate can be calculated using the formula [28]: inhibitory rate (%) = 100% * ((colony diameter in control—disc diameter)—(colony diameter in treatment—disc diameter)) / (colony diameter in control—disc diameter).

## Results

### Taxonomic identification and characteristics of extracellular enzyme production

Total of 209 bacterial strains were isolated from the rhizosphere soil of peppers, among which 23 strains exhibited antagonistic effects against *P*. *capsici*, including Pseudomonas, Bacillus and Burkholderia.

**Fig 1:**
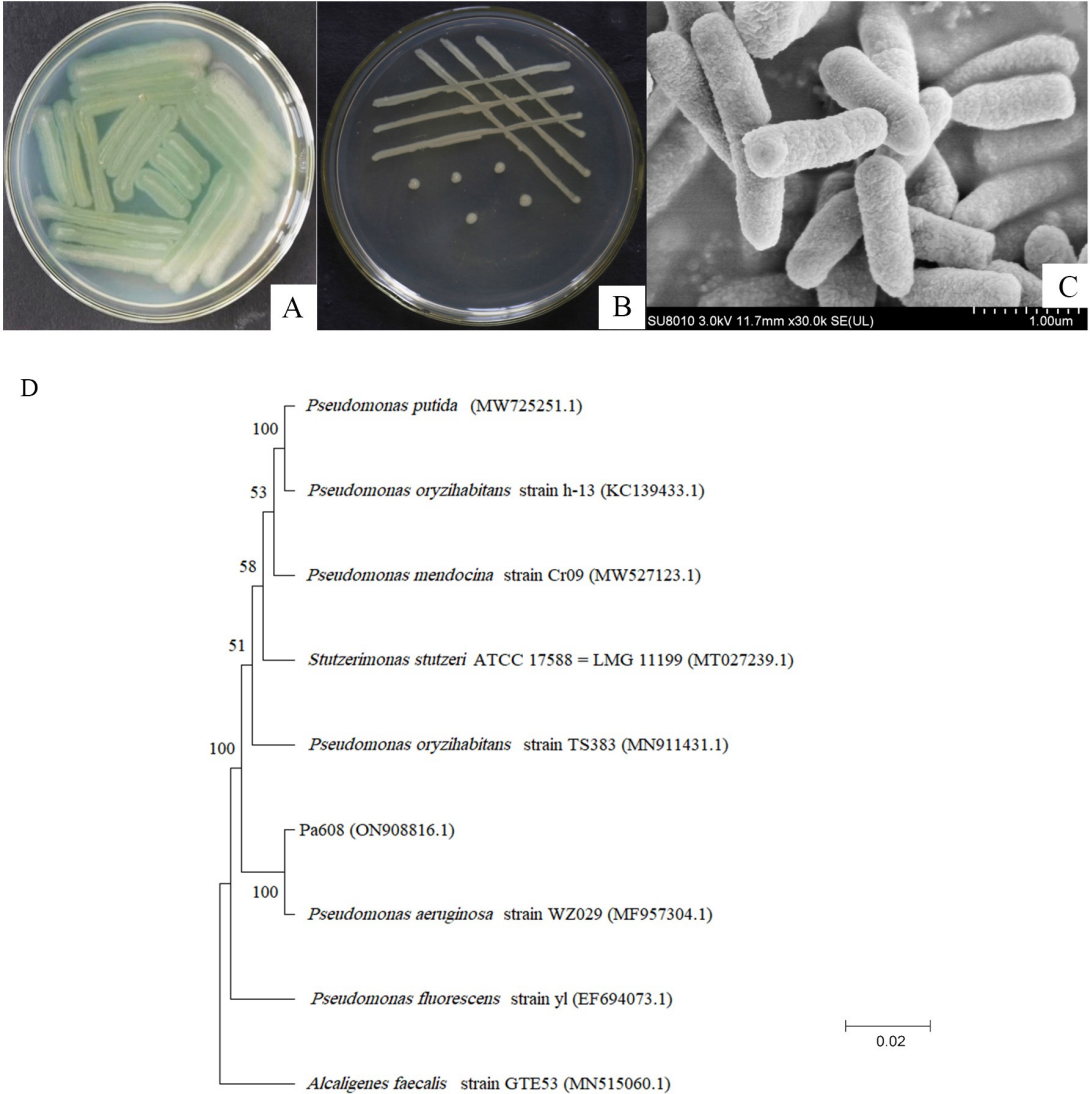
Taxonomic identification of the strain Pa608. A, Colony on NA medium. B, Colony on LB medium. C, Morphology. D, Phylogenetic tree.

The strain with the largest inhibition zone was designated as Pa608. After a two-day incubation period at 30°C on an NA plate, the strain Pa608 exhibited green colonies (Fig 1A), while on an LB plate, strain Pa608 showed white, smooth colonies without visible pores (Fig 1B). The Gram staining results were negative. Scanning electron microscopy observations displayed rod-shaped cells measuring approximately 1–5 μm in length and 0.5–1.0 μm in width (Fig 1C). A phylogenetic tree of 16S rRNA sequences showed that the *P*. *aeruginosa* WZ2029 was in the same clade as the strain Pa608 with a percent identity of 100.00% (Fig 1D), suggesting that they were closely related. Based on morphological characteristics and 16S rRNA sequences, the strain Pa608 was identified as *P*. *aeruginosa*.

The strain Pa608 can produce protease and cellulose, forming transparent zones (S1A and S1B Fig), however, no amylase secretion was detected (S1C Fig). Moreover, it demonstrated phosphate-solubilizing ability by forming a phosphorus-solubilizing circle (S1D Fig).

### Inhibition effect of the strain Pa608 on *P*. *capsici* and other plant pathogens

The strain Pa608 demonstrated significant suppression of mycelial growth in *P*. *capsici*, forming a marked inhibition zone (S2A Fig). Furthermore, it exhibited inhibitory effects to varying degrees on the growth of *S*. *sclerotiorum*, *P*. *oryzae*, *Diaporthe citri*, *B*. *cinerea*, *F*. *graminearum*, and *P*. *simplicissimum* (S2B–S2G Fig).

### Pot experiment

**Fig 2:**
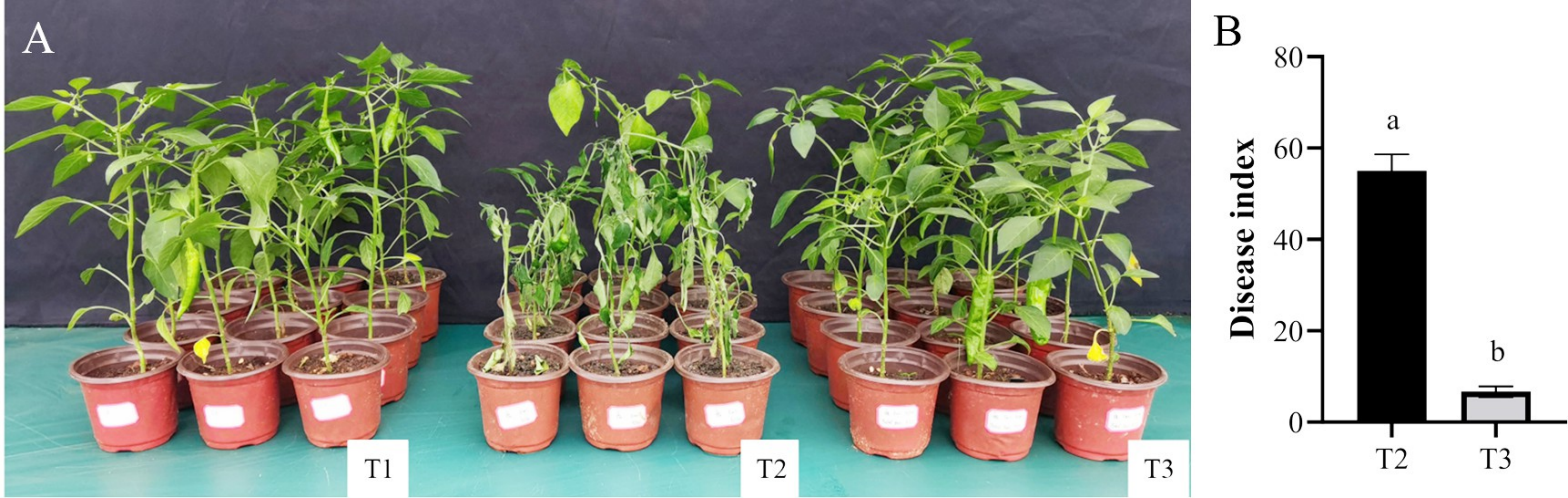
Pot Experiment. A, Growth of pepper plants in pots. T1 represented treatment with sterilized water; T2 represented treatment with zoospores of *P*. *capsici*; T3 represented treatment with zoospores of *P*. *capsici* and the strain Pa608 bacterial suspension. B, Disease index. Different letters indicated significant differences (*p* < 0.05) between treatments according to Duncan’s multiple-range test following one-way ANOVA.

At 45 days after treatment, a significant portion of the pepper plants in the T2 group wilted, with incidence of 100% (Fig 2A). Conversely, pepper plants in the T3 group showed no obvious symptoms, resulting in incidence of 12%. The disease index of T2 at 55 was markedly higher than that of T3 at 6.6 (Fig 2B). The control efficiency reached 88.0%, indicating the effective suppression of *P*. *capsici* by the strain Pa608.

### Population dynamic of the strain Pa608 in pepper rhizosphere

**Fig 3:**
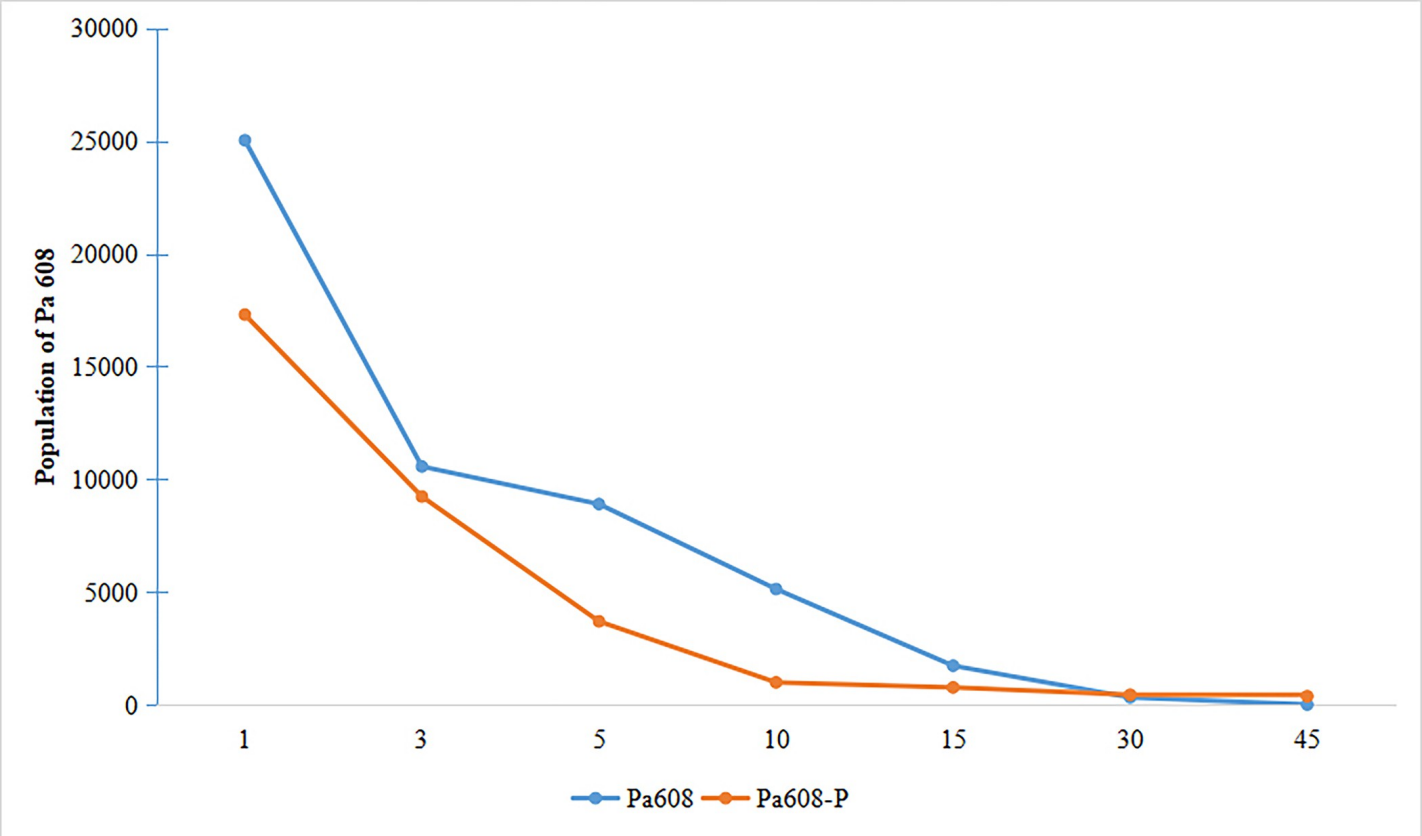
Population dynamic of the strain Pa608. The "Blue line + Pa608" represented the inoculation of the strain Pa608 alone, while the "Blue line + Pa608-P" represented the inoculation of the strain Pa608 and *P*. *capsici*.

Within the first 15 days after inoculation, the population of the strain Pa608 in the pepper rhizosphere soil rapidly decreases. From day 15 to day 45, the population of the strain Pa608 declines slowly and tends to stabilize (Fig 3). After inoculation with the strain Pa608 alone, the population of Pa608 was slightly higher than the population of Pa608 in the rhizosphere when co-inoculated with *P*. *capsici*. This indicates that the strain Pa608 was capable of colonizing the pepper rhizosphere.

### Field experiment

**Fig 4:**
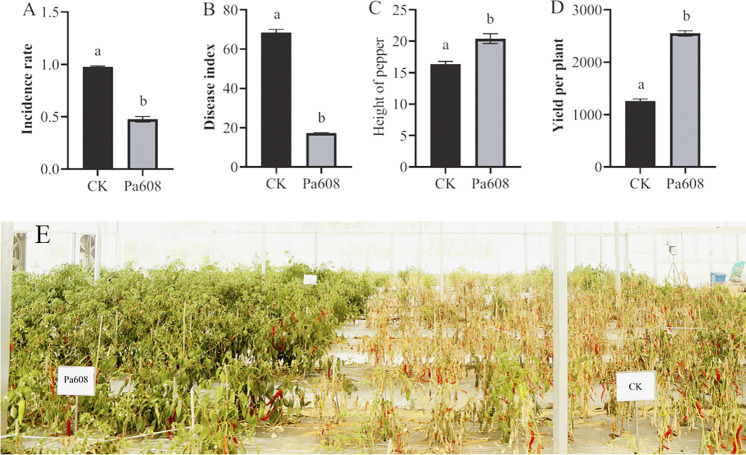
Field experiment. A, Incidence rate. B, Disease index. C, Height of pepper plants. D, Yield per plant. E, Field performance of pepper plants. Different letters indicated significant differences (*p* < 0.05) between treatments according to Duncan’s multiple-range test following one-way ANOVA.

Following the application of the strain Pa608 bacterial suspension, the incidence rate for Treatment 2 was 48.9%, with disease index of 17.3. The rate was notably lower than that of the control group (Fig 4A and 4B), resulting in control efficiencies of 74.9%. Moreover, the strain Pa608 demonstrated a growth-promoting effect on pepper plants, as seen in the significantly greater height of plants (19.96 cm) and yield (2611.02 g per plant) in Treatment 2 compared to the control group (15.90 cm and 1209.71 g per plant) (Fig 4C and 4D). Furthermore, pepper plants treated with the strain Pa608 exhibited better health and lower mortality rates compared to those in the control group (Fig 4E), where most pepper plants perished.

### GC-MS analysis

The GC-MS analysis data of the sterile filtrate of the strain Pa608 revealed a total of 51 secondary metabolites with a similarity of over 70% (S1 Table). These substances were mainly categorized as alcohols, alkanes, ketones, esters, sesquiterpenes, and phenazines. Among them, 3-carene exhibited a matching factor as high as 88.3% with a peak area of 1,367,054.6. The α-pinene had a matching factor of 86.85% and a peak area of 1,427,453.5 in the detected substances.

### Biocontrol activity assessment of α-pinene and 3-carene

**Fig 5:**
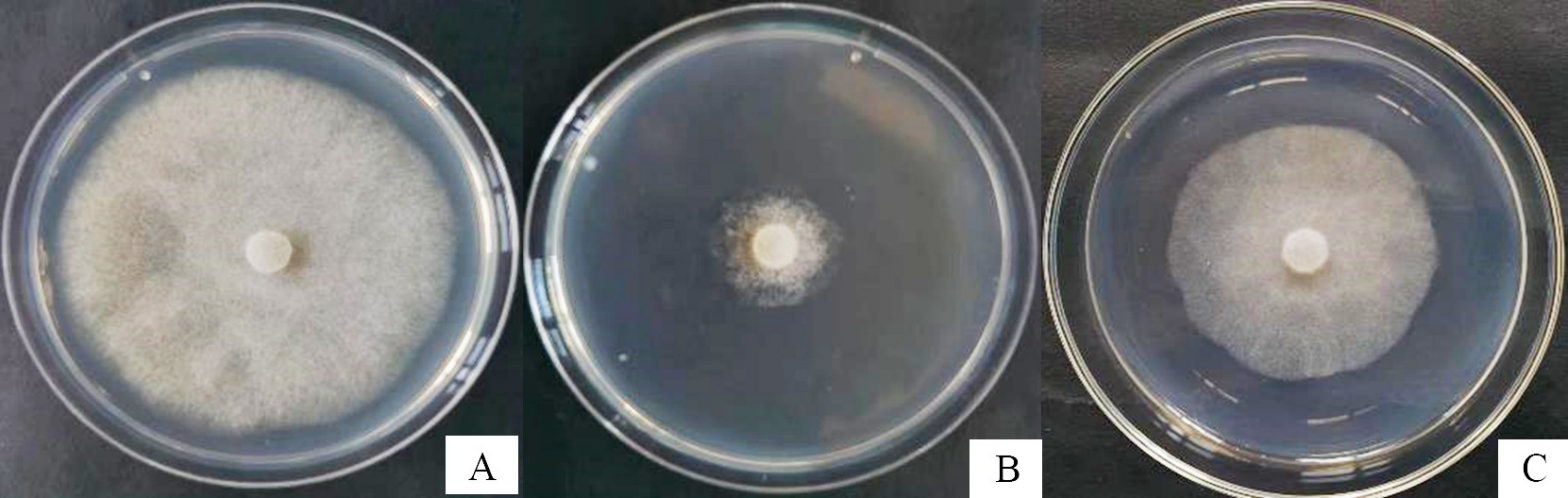
Inhibition of *P*. *capsici* growth by α-pinene and 3-carene. A, *P*. *capsici* in the control group. B, *P*. *capsici* treated with α-pinene. C, *P*. *capsici* treated with 3-carene.

The 3-carene and α-pinene exhibit anti-oomycetes activity against *P*. *capsici*. Compared to *P*. *capsici* in the control group (Fig 5A), α-pinene inhibits *P*. *capsici* by 84.9% at a concentration of 5 mg/L (Fig 5B), whereas 3-carene only achieves a 35.2% inhibition rate against the pathogen at the same concentration (Fig 5C).

## Discussion

### The inhibitory activity of the strain Pa608 against *P*. *capsici*

The utilization of soil-borne biocontrol microorganisms in the rhizosphere to suppress soil-borne pathogens is a significant research focus in sustainable agriculture, benefiting from ample available resources [29]. In our investigation, the *P*. *aeruginosa* strain Pa608 exhibited notable inhibitory activity against *P*. *capsici* both *in vitro* and *in vivo*, consistent with previous findings on Pseudomonas in combating *P*. *capsici* [30,31]. Pseudomonas exhibits a broad antimicrobial spectrum, capable of inhibiting a diverse range of plant pathogens, including fungi and oomycetes. Particularly, destructive species like *P*. *cactorum*, *P*. *capsici*, and *P*. *infestans* can also be suppressed by certain Pseudomonas species [32–34]. In our research, we observed suppression of mycelial growth by the strain Pa608, consistent with findings from previous studies [16,17]. However, the investigation did not include an assessment of the suppression of zoospore germination and germ tube elongation by the strain Pa608. It was possible to speculate that the strain Pa608 may also inhibit the germination of motile spores and germ tube elongation of Phytophthora in peppers, as strains of the same *P*. *aeruginosa* species have demonstrated similar activities [16,17].

Our pot experiment showed that the strain Pa608 exhibited a high control efficiency against pepper Phytophthora blight at 88.0%, surpassing the control efficiency of 73.1% achieved by *Streptomyces olivaceus* [35] and the 77% control efficiency reported for *P*. *lini* [36]. Furthermore, the control efficiency in field trials by the strain Pa608 was 74.9%, equivalent to the field efficacy (75.16%) of the combination of XJC2-1 and the fungicide dimethomorph [37], suggesting its potential in field management. Moreover, the high control efficiency also reflected the stability of strain Pa608 in the pepper rhizosphere. However, the control efficiency of the strain Pa608 was lower than that of the Bacillus mixture at 88.0% [38]. This indicates that multi-species combinations of microorganisms demonstrate higher efficacy in biological control compared to single strains. The trend in the development of biological control is to utilize artificially synthesized microbial communities to fully leverage the advantages of diverse microbial combinations, continuously and effectively exerting biological control [39]. In this study, we successfully isolated Bacillus and Burkholderia from the rhizosphere of peppers. The next step involves screening these bacteria to construct a microbial community for biological control centered around the strain Pa608.

### Colonization characteristic of the strain Pa608 in pepper rhizosphere and their growth-promoting features

The effectiveness of biological control primarily depends on the population density of biocontrol microorganisms that can easily colonize the plant rhizosphere and reproduce quickly, enabling them to exert long-lasting control effects [40]. Consequently, biocontrol microorganisms with wide adaptability and easy colonization have become the preferred choice for researchers in plant protection. Pseudomonas spp. are widely distributed in the environment and have developed adaptive mechanisms to colonize a wide range of ecological niches, such as animal hosts, water environments and rhizosphere [41]. In particular, in the plant rhizosphere, Pseudomonas can thrive by utilizing plant exudates, leading to their abundant proliferation. The strain Pa608 was isolated from pepper rhizosphere soil, demonstrating their colonization in soil. Moreover, the results of detection confirmed that the strain Pa608 could survive in the rhizosphere soil for up to 45 days. However, the population of the strain Pa608 declined after inoculation and stabilized at a later period, possibly due to the following reasons: under natural conditions, the number of Pa608 present in the soil was significantly lower than the number introduced through inoculation, resulting in the death of excess Pa608 due to limited nutrients. In the later stages of pepper growth, as pepper roots proliferate and exudates increase, they could nourish Pa608 and sustain their population size.

The strain Pa608 demonstrated a growth-promoting effect on pepper plants, as evidenced by the significantly greater plant height and yield. Most biocontrol Pseudomonas species exhibit plant growth promotion activity, with mechanisms including phosphate solubilization [42] and production of indole acetic acid [43]. We found that the strain Pa608 possessed phosphate-solubilizing capability, which facilitates the dissolution of phosphorus, aiding in its uptake by peppers and indirectly promoting pepper growth. The production of indole acetic acid by the strain Pa608 needs further verification.

### Anti-oomycete activity of low molecular weight substances with volatility produced by the strain Pa608

*P*. *aeruginosa* is known to produce various secondary metabolites that play a crucial role in its virulence and interactions with other organisms [44]. In our study, the strain Pa608 produced many secondary metabolites, including alcohols, alkanes, ketones, esters, sesquiterpenes, and phenazines. Different strains of *P*. *aeruginosa* may also produce similar substances, but there may be differences in the types and amounts. The types and quantities of secondary metabolites produced by *P*. *aeruginosa* are related to the genetic characteristics of the strain, the type of culture medium, and environmental conditions.

Phenazine compounds are redox-active nitrogen-containing heterocyclic molecules that exhibit broad-spectrum antibiotic activity against various fungal, bacterial, and oomycete plant pathogens [45–48]. For instance, phenazine-1-carboxylic acid, which is secreted by the *P*. *aeruginosa* strain GC-B26, has been shown to inhibit the growth of both *P*. *capsici* and *C*. *orbiculare* [31]. In this research, 1,6-Dimethylphenazine and 1-Hydroxy-6-methylphenazine was detected from fermentation broth of the strain Pa608, indicating that *P*. *capsici* itself may produce certain types of phenazine compounds.

The focus of our research was on low molecular weight substances with volatility, diverging from previous studies that centered on phenazines and cyclic lipopeptides [49]. 3-carene is low molecular weight substance, possessed volatility and antimicrobial properties [50], which exhibited anti-oomycetes to some extent in our research. Particularly, α-pinene, secreted by the strain Pa608, demonstrated significant anti-oomycete activity with an 84.9% inhibition. Previous research has indicated that Burkholderia tropica produced α-pinene, which inhibits the growth of fungal pathogens such as *Colletotrichum gloeosporioides*, *F*. *culmorum*, *F*. *oxysporum*, and *Sclerotium rolfsii* and destructs fungal hyphae [51], suggesting the potential of α-pinene in developing novel fungicides.

## Conclusion

Taken together, the strain *P*. *aeruginosa* Pa608 exhibits strong inhibitory activity against *P*. *capsici*, significantly reducing the pot and field incidence rate and increasing pepper height and yield. In particular, this strain can produce α-pinene, effectively inhibiting the growth of *P*. *capsici*. The next step is to further explore the mechanism of α-pinene against *P*. *capsici* and the synthesis pathway of α-pinene in the strain Pa608.

## Supporting information

S1 Table Major substances in ethyl acetate extracts. (DOCX)

S1 Fig Characteristics of extracellular enzyme production. A, protease. B, cellulose. C, amylase. D, phosphorylase.
(TIF)

S2 Fig Inhibition effect of the strain Pa608 on some pathogens. A, *P*. *capsici*. B, *S*. *sclerotiorum*. C, *P*. *oryzae*. D, *Diaporthe citri*. E, *B*. *cinerea*. F, *F*. *graminearum*. G, *P*. *simplicissimum*.
(TIF)
