# Supplementary material for: Evaluation of biocontrol efficacy of rhizosphere Pseudomonas aeruginosa for management of Phytophthora capsici of pepper
Source: PLoS One. 2024 Sep 20;19(9):e0309705. doi: 10.1371/journal.pone.0309705 (PMC11414977; doi:10.1371/journal.pone.0309705)
Supplement: S1 Table — (DOCX) [file pone.0309705.s001.docx]

**S1 Table. Major substances in ethyl acetate extracts**

| Substance Name | CAS No. | Molecular Formula | Group Area | Matching factor |
| --- | --- | --- | --- | --- |
| Butanoic acid, 3-methyl- | 503-74-2 | C_5_H_10_O_2_ | 48162344.8 | 93.5 |
| Endo-3-acetamidocamphor | 3750-49-0 | C_11_H_19_NO | 18230267.9 | 70.3 |
| Cyclononasiloxane, octadecamethyl- | 556-71-8 | C_18_H_54_O_9_Si_9_ | 7865359.3 | 74.9 |
| Cyclodecasiloxane, eicosamethyl- | 18772-36-6 | C_20_H_60_O_10_Si_10_ | 6315379.1 | 73.7 |
| 1H-Quinolin-4-one, 2-octyl- | 80554-60-5 | C_17_H_23_NO | 5181707.6 | 70.0 |
| (+)-Dibenzoyl-L-tartaric acid anhydride | 64339-95-3 | C_18_H_12_O_7_ | 5102251.5 | 75.6 |
| Dodecane, 1-iodo- | 4292-19-7 | C_12_H_25_I | 4586388.8 | 71.5 |
| 2-Bromo dodecane | 13187-99-0 | C_12_H_25_Br | 4512287.4 | 74.1 |
| Bicyclo[3.1.1]heptane, 6,6-dimethyl-2-methylene-, (1S)- | 18172-67-3 | C_10_H_16_ | 3775119.5 | 85.1 |
| Dodecane, 4,6-dimethyl- | 61141-72-8 | C_14_H_30_ | 3464213.4 | 82.7 |
| Decane, 3,8-dimethyl- | 17312-55-9 | C_12_H_26_ | 3380995.1 | 79.8 |
| Cyclodecasiloxane, eicosamethyl- | 18772-36-6 | C_20_H_60_O_10_Si_10_ | 3081360.9 | 71.5 |
| Hexadecane, 2,6,10,14-tetramethyl- | 638-36-8 | C_20_H_42_ | 2948199.1 | 71.6 |
| Heneicosane | 629-94-7 | C_21_H_44_ | 2882094.4 | 70.8 |
| Heptasiloxane, hexadecamethyl- | 541-01-5 | C_16_H_48_O_6_Si_7_ | 2862043.7 | 75.7 |
| Heptacosane | 593-49-7 | C_27_H_56_ | 2584344.8 | 72.1 |
| Cyclononasiloxane, octadecamethyl- | 556-71-8 | C_18_H_54_O_9_Si_9_ | 2533710.1 | 79.5 |
| Cyclododecanol | 1724-39-6 | C_12_H_24_O | 2465973.4 | 72.8 |
| 2-Bromo dodecane | 13187-99-0 | C_12_H_25_Br | 2450454.9 | 82.2 |
| Hexadecane | 544-76-3 | C_16_H_34_ | 2422763.9 | 77.4 |
| Nonane, 2,2,4,4,6,8,8-heptamethyl- | 4390-04-9 | C_16_H_34_ | 2035108.2 | 73.7 |
| Undecane, 3,8-dimethyl- | 17301-30-3 | C_13_H_28_ | 1979189.8 | 80.1 |
| Tetradecane, 4-ethyl- | 55045-14-2 | C_16_H_34_ | 1855609.0 | 73.3 |
| Oxirane, tridecyl- | 18633-25-5 | C_15_H_30_O | 1776402.1 | 75.2 |
| Isobutyl acetate | 110-19-0 | C_6_H_12_O_2_ | 1568734.8 | 86.8 |
| Nonane, 2,2,4,4,6,8,8-heptamethyl- | 4390-04-9 | C_16_H_34_ | 1485508.9 | 71.9 |
| Heptasiloxane, hexadecamethyl- | 541-01-5 | C_16_H_48_O_6_Si_7_ | 1484717.8 | 73.5 |
| (1R)-2,6,6-Trimethylbicyclo[3.1.1]hept-2-ene | 7785-70-8 | C_10_H_16_ | 1427453.5 | 86.8 |
| Cyclohexanone | 108-94-1 | C_6_H_10_O | 1396932.0 | 81.6 |
| Cobalt, bis(.eta.-5-piperidinylcyclopentadienyl)- | 1000162-04-6 | C_20_H_28_CoN_2_ | 1391998.7 | 71.7 |
| 3-Carene | 13466-78-9 | C_10_H_16_ | 1367054.6 | 88.3 |
| Cycloheptasiloxane, tetradecamethyl- | 107-50-6 | C_14_H_42_O_7_Si_7_ | 1344741.1 | 70.3 |
| Heptasiloxane, hexadecamethyl- | 541-01-5 | C_16_H_48_O_6_Si_7_ | 1304602.2 | 76.8 |
| Hexadecane | 544-76-3 | C_16_H_34_ | 1244874.0 | 72.9 |
| Undecane, 4-ethyl- | 17312-59-3 | C_13_H_28_ | 1008980.2 | 72.8 |
| Cyclononasiloxane, octadecamethyl- | 556-71-8 | C_18_H_54_O_9_Si_9_ | 990704.7 | 81.4 |
| Phthalic acid, heptyl tridec-2-yn-1-yl ester | 1000315-44-0 | C_28_H_42_O_4_ | 881756.0 | 77.1 |
| Octane, 3,5-dimethyl- | 15869-93-9 | C_10_H_22_ | 841410.3 | 83.7 |
| 2(5H)-Furanone, 5,5-dimethyl- | 20019-64-1 | C_6_H_8_O_2_ | 824939.4 | 81.3 |
| Octane, 4-ethyl- | 15869-86-0 | C_10_H_22_ | 804369.5 | 80.9 |
| Pentane, 3-ethyl-3-methyl- | 1067-08-9 | C_8_H_18_ | 720476.2 | 71.4 |
| 3-Ethyl-3-methylheptane | 17302-01-1 | C_10_H_22_ | 686328.5 | 77.9 |
| Tetracosamethyl-cyclododecasiloxane | 18919-94-3 | C_24_H_72_O_12_Si_12_ | 679438.2 | 76.9 |
| Benzyl chloride | 100-44-7 | C_7_H_7_Cl | 629227.5 | 77.5 |
| 4,4-Dimethyl octane | 15869-95-1 | C_10_H_22_ | 574209.7 | 76.4 |
| Benzene, 1-methyl-3-(1-methylethyl)- | 535-77-3 | C_10_H_14_ | 572399.6 | 72.6 |
| o-Cymene | 527-84-4 | C_10_H_14_ | 569003.2 | 77.4 |
| Decane, 2,4-dimethyl- | 2801-84-5 | C_12_H_26_ | 566779.2 | 73.0 |
| CH_3_C(O)OCH(CH_3_)C(O)CH_3_ | 4906-24-5 | C_6_H_10_O_3_ | 559036.2 | 71.2 |
| 1-Hydroxy-6-methylphenazine | 14031-08-4 | C_13_H_10_N_2_O | 2249120.5 | 59.9 |
| 1,6-Dimethylphenazine | 58718-43-7 | C_14_H_12_N_2_ | 12181675.2 | 60.5 |
