# Supplementary figures and images for: Evaluation of biocontrol efficacy of rhizosphere Pseudomonas aeruginosa for management of Phytophthora capsici of pepper
Source: PLoS One. 2024 Sep 20;19(9):e0309705. doi: 10.1371/journal.pone.0309705 (PMC11414977; doi:10.1371/journal.pone.0309705)

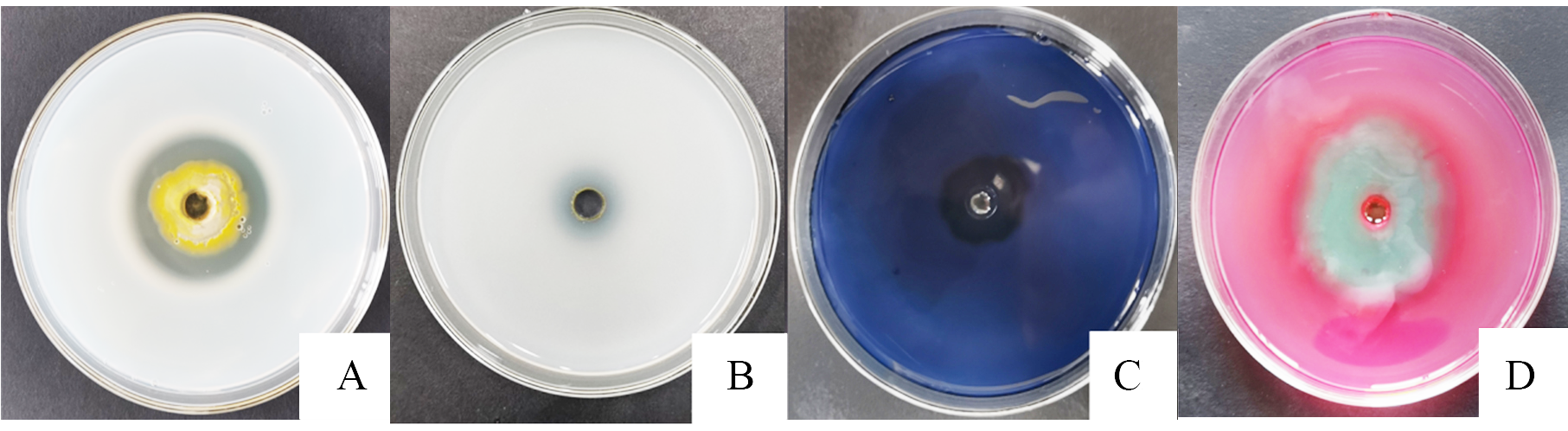

Supplement: S1 Fig — A, protease. B, cellulose. C, amylase. D, phosphorylase. (TIF) [file pone.0309705.s002.tif]

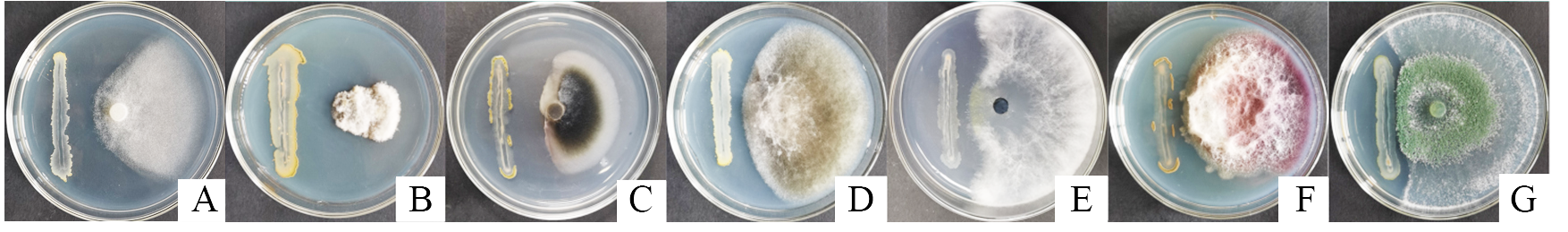

Supplement: S2 Fig — A, P. capsici. B, S. sclerotiorum. C, P. oryzae. D, Diaporthe citri. E, B. cinerea. F, F. graminearum. G, P. simplicissimum. (TIF) [file pone.0309705.s003.tif]
